# Evaluation of *Trichoderma* spp., *Pseudomonas* *fluorescens* and *Bacillus subtilis *for biological control of Ralstonia wilt of tomato

**DOI:** 10.12688/f1000research.12448.3

**Published:** 2018-03-22

**Authors:** Shiva Yendyo, Ramesh G.C., Binayak Raj Pandey

**Affiliations:** 1 Kishan Call Center, Bharatpur-4, Chitwan, 44207, Nepal; 2 Department of Quality Control, Agricare Nepal Pvt. Ltd., Bharatpur-4, Chitwan, 44207, Nepal

**Keywords:** Trichoderma, Pseudomonas fluorescence, Bacillus subtilis, Ralstonia solanacearum, biocontrol agent, bio-efficacy, bacterial wilt, tomato

## Abstract

**Background:** *Ralstonia *spp. is a major pathogenic microbe for tomato, which invades the roots of diverse plant hosts and colonizes xylem vessels causing wilt, especially in tropical, subtropical and warm-temperate regions. *Ralstonia *spp. **produces several virulence factors helping it to invade the plant’s natural defense mechanism. Native isolates of *Trichoderma spp., Pseudomonas fluorescens* and *Bacillus subtilis* can be used as biocontrol agents to control the bacterial wilt and combined application of these beneficial microbes can give better results.

**Methods: **Bacterial wilt infection in the field was identified by field experts and the infected plant part was used to isolate *Ralstonia *spp. **in CPG media and was positively identified. Subsequently, the efficacy of the biocontrol agents was tested and documented using agar well diffusion technique and digital microscopy. 2ml of the microbial concentrate (10 ^9^ cells/ml) was mixed in one liter of water and was applied in the plant root at the rate of 100 ml per plant as a treatment method.

**Results: **It was observed that the isolated *Trichoderma* spp. AA2 and *Pseudomonas fluorescens *PFS were most potent in inhibiting the growth of *Ralstonia *spp. *, *showing ZOI 20.67 mm and 22.33 mm, respectively. Digital microscopy showed distinct inhibitory effect on the growth and survival of *Ralstonia *spp *. *The results from the field data indicated that *Trichoderma* spp. and *Pseudomonas fluorescens *alone were able to prevent 92% and 96% of the infection and combination of both were more effective, preventing 97% of infection. Chemical control methods prevented 94% of infection. *Bacillus subtilis* could only prevent 84 % of the infection.

**Conclusions: **Antagonistic effect against *Ralstonia spp.* shown by native isolates of *Trichoderma* spp. and *P. fluorescens* manifested the promising potential as biocontrol agents. Combined application gave better results. Results shown by *Bacillus subtilis* were not significant.

## Author endorsement

Dr. Sushil Thapa confirms that the author has an appropriate level of expertise to conduct this research, and confirms that the submission is of an acceptable scientific standard. Dr. Sushil Thapa declares he has no competing interests. Affiliation: Texas A&M AgriLife Research, Amarillo, TX 79106, USA.

## Introduction

Bacterial wilt caused by *Ralstonia spp.* is a major pathogenic microbe for tomatoes ( *Solanum lycopersicum* L). It invades the roots of diverse plant hosts from the soil and aggressively colonizes the xylem vessels, causing bacterial wilt disease ^1– 3^. It is a devastating plant disease most commonly found in tropical, subtropical and warm-temperate regions ^4, 5^. *Ralstonia* spp. produces several known virulence factors, including extracellular polysaccharide (EPS), and a combination of plant cell wall-degrading enzymes, such as endoglucanase (EG) and polygalacturonase (PG). Mutants lacking EPS and EG shows reduced virulence ^6– 8^. The major virulence factors for this pathogen are plant cell wall-degrading polygalacturonases (PGs) ^9^.

Various biocontrol agents are used to control the bacterial wilt caused by *Ralstonia* spp. *Trichoderma harzianum* ^10, 11^, *Trichoderma viride* ^12^, *Trichoderma asperellum* ^13^, *Trichoderma virens* ^14^, *Pseudomonas fluorescens* ^15, 16^ and *Bacillus subtilis* ^17^ are used as biocontrol agents to control bacterial wilt. Combination treatment methods using two or more of these agents are more effective in managing the disease than treatment using a single biocontrol agent ^10, 18, 19^. Chemical bactericides and fungicides induce resistance in pathogens during long-term use, which ultimately makes the pathogen tolerant to these chemical applications ^20– 23^. Hence, there should be a focus on the use of biological methods to control plant disease.

This study focuses on evaluating the efficacy of different native isolates of *Trichoderma* species, *B. subtilis* and *P. fluorescens* against bacterial wilt disease caused by the pathogen *Ralstonia* spp. in the tomato plant. The study hypothesizes that the native isolates of *Trichoderma* spp., *B. subtilis* and *P. fluorescens* can be used as bioantagonistic agents to control bacterial wilt ( *Ralstonia* spp.) of tomato. This study tries to establish the hypothesis by the microscopic examinations, agar well diffusion technique and field trials in infected tomato plots, by calculating their efficacy and comparing them with chemical methods of treatment.

## Methods

### Selection of the study plot

The sample collection and field trials were done at Agro Narayani Farm, Sukranagar, Chitwan District, Nepal (Latitude: 27.582016 and Longitude: 84.272259) where the bacterial wilt infection was recorded in the previous harvest (done 3 months before the test). Also, *Ralstonia* spp. were observed in the Casamino acid-Peptone-Glucose (CPG) Agar plates from the soil samples. The tests in the field were conducted from 11 March 2017 to 8 July 2017 for 120 days in new transplants. At the time of transplant, compost fertilizer at the rate 2.94 kg/m ^2^, urea at the rate of 23.59 gm/m ^2^, potash at the rate of 29.49 gm/m ^2^, DAP 19.66 gm/m ^2^, borax at the rate 1.97 gm/m ^2^ and zinc at the rate 1.97 gm/m ^2^ were applied. At 45 days and 90 days of transplant NPK 20:20:20 was applied at the rate 9.83/m ^2^. The spacing of the plant was 50 cm in double row system of 50 × 50 cm. The plot size was 50 m ^2^ and total of 8 plots were used having 100 plants per plot. Weather data were also collected from online resources as a reference.

### Identification of bacterial wilt infection in the field

Physical symptoms, such as wilting of young leaves, discolored tissue at the dissected part of the stem base, and white, slimy ooze when the dissected part of the plant was kept in the glass of water, were used for the identification of infected plants ^24^.

### Isolation and identification of *Ralstonia* spp.

Bacterial wilt infection in tomato plants ( *Solanum lycopersicum* L. var. Manisha) grown in Chitwan, Nepal, was positively identified by S. Yendyo of Kishan Call Center (KCC). Six whole plants were brought to the Quality Control laboratory of Agricare Nepal Pvt. Ltd. in a sterile bag for the isolation of the pathogen.

*Ralstonia* spp. was isolated from dissected sections of the infected tomato plants on Casamino acid-Peptone-Glucose (CPG) Agar (casein hydrolysate 1 g/l, peptone 10 g/l, glucose 5g/l, agar 15g/l) which is preferred media for isolation of *Ralstonia* spp. The stems of the infected plant were washed three times with autoclaved distilled water and then blot dried. After drying the stem were washed with 80% ethanol solution, then 1% sodium hypochlorite (NaOCl) solution was applied for 2 minutes. Final washing was done with autoclaved distilled water three times. The xylem of 2–3 cm from the stem was dissected and the sap was rubbed in CPG medium and inoculated for 48h at 28°C. Identification was done on the basis of the morphology of the colony on CPG medium, Gram staining and microscopic examination ^25, 26^.

### Isolation of antagonists

A total of 13 strains were used in this study (see Table 1). Six strains of *Trichoderma spp.*, two strains of *Pseudomonas fluorescens* and one strain of *Bacillus subtilis* (GenBank Accession No. MG952584) were isolated from soils and root soils collected from different sites in Nepal. ATCC 13525 strain of *P. fluorescens* from Microbiologics, St. Cloud, MN 56303, France was used as the reference for isolated *Pseudomonas* species. *Trichoderma harzianum*, *Trichoderma virens* and *Trichoderma asperellum* strains were provided by Tamil Nadu Agricultural University (TNAU), Coimbatore, Andhra Pradesh, India, and were used as reference species for isolated *Trichoderma* spp for two purposes. First, the reference species were used to identify the isolated species through morphological and microscopic analysis and second, the reference species were used to compare the antagonistic effects exhibited by the native isolates. All the species were collected from the tropical region except *Bacillus subtilis* which was collected from mid hill regions of Nepal. The reason behind this was that the site from where the samples were collected was unaffected by the wilt disease compared to nearby sites where the bacterial wilt was severely present. The testing of the soil samples from the unaffected site revealed the substantial presence of *Bacillus subtilis* which may have induced systemic resistance in the plants.

**Table 1.** Biocontrol agents used to analyze bio-efficacy against *Ralstonia* spp. This includes the shortcode given to the isolates for identification in this study.

Name of strains and source	Source	Code given
*Trichoderma* spp.	*Parthenium hysterophorus* L. rhizoplane soil	AA2
*Trichoderma* spp.	*Cannabis sativa* L. rhizoplane soil	AG3
*Trichoderma* spp.	*Solanum viarum* Dunal rhizoplane soil	AKD
*Trichoderma* spp.	Agricare Nepal Pvt. Ltd. top soil	A5
*Trichoderma* spp.	*Brassica juncea* L. spp. rhizoplane soil	A9
*Trichoderma* spp.	Chitwan National Park top soil	A10
*Trichoderma harzianum*	TNAU, India	ATH
*Trichoderma virens*	TNAU, India	ATV
*Trichoderma asperellum*	TNAU, India	ATA
*Bacillus subtilis* ( MG952584)	Bhaktapur top soil	BS
*Pseudomonas fluorescens*	*Parthenium hysterophorus* L. rhizoplane soil	PFS
*Pseudomonas fluorescens*	*Chenopodium album* L. rhizoplane soil	PFB
*Pseudomonas fluorescens*	ATCC 13525	PFA

*Trichoderma spp*. were isolated by serial dilution method using TSM agar plate (K _2_HPO _4_: 0.9 g/l, MgSO _4_: 0.2 g/l, KCl: 0.15 g/l, NH _4_Cl: 1.05 g/l, Glucose 3 g/l, Rose Bengal: 0.15 g/l, agar 20 g/l, streptomycin: 100 mg/l, tetracycline: 50 mg/l) ^27^. 1 gm of each soil samples were suspended in 9 ml of sterile distilled water and vortexed (Accumax India, New Delhi-110058, India) for 5 min. The soil suspension was then serially diluted to 10 ^-3^ and 10 ^-4^. Pour plate technique was used by mixing 1 ml of the diluted soil suspension in 3 TSM agar plates for each sample and incubated at 25°C for 5 days. The strains were purified on TSM agar plates using sub-culture technique.

*Pseudomonas fluorescens* were isolated by serial dilution method using King’s B agar (Peptone: 20g/l, K _2_HPO _4_: 1.5 g/l, MgSO _4_: 1.5 g/l, glycerol: 10 ml/l, agar: 20g/l) ^28^. 1 gm of each soil samples were suspended in 9 ml of sterile distilled water and vortexed (Accumax India, New Delhi-110058, India) for 5 min. The soil suspension was then serially diluted to 10 ^-6^. Pour plate technique was used by mixing 1 ml of the diluted soil suspension in 3 King’s B agar plates for each sample and incubated at 27°C for 48 h and the fluorescence was observed in UV light. The fluorescent strains were purified on King’s B agar plates using sub-culture technique.

*Bacillus subtilis* was isolated by serial dilution method using Nutrient Agar (Peptic digest of animal tissue: 5 g/l, NaCl: 5 g/l, beef extract: 1.5 g/l, yeast extract: 1.5 g/l, agar: 20g/l) ^29^. 1 gm of each soil samples were suspended in 9 ml of sterile distilled water and vortexed (Accumax India, New Delhi-110058, India) for 5 min. The soil suspension was then serially diluted to 10 ^-6^. Pour plate technique was used by mixing 1 ml of the diluted soil suspension in 3 NA plates for each sample and incubated at 27°C for 48 h. The strains were purified on NA agar plates by using sub-culture technique.

### Evaluating the efficacy of antagonists against *Ralstonia* spp.

All thirteen isolates were screened against *Ralstonia* spp *.* by agar well diffusion technique ^30^. *Ralstonia* spp *.* on CPG agar plates were transferred to the nutrient broth and shaken in a rotary shaker (Talboys, Henry Troemner, LLC, USA) at 100 rpm at 27°C for 24 h. Similarly, the TSM, King’ B and NB were prepared for all *Trichoderma* spp., *P. fluorescens* and *B. subtilis,* respectively, and incubated for 7 days, 48 h and 48h, respectively. After incubation of the antagonists, 5 ml of broth suspension were centrifuged at 5000 rpm for 5 min and the supernatant was stored at 4°C for further procedure. Then, *Ralstonia* spp *.* suspension of 10 ^8^ cells/ml was prepared as per McFarland 0.5 turbidity method ^31^ and was swabbed on NA plates. Holes of 5 mm were punched into the agar plate and 40 µl of the supernatant prepared were added separately and the plates were incubated at 27°C for 48 h. Inhibition of *Ralstonia* spp *.* growth was assessed by measuring the radius in mm of the zone of inhibition (ZOI) after incubation.

For microscopic visualization of the inhibition, CPG agar plates were prepared to provide the most favorable growth to *Ralstonia* spp. *,* and the respective 5 mm mycelial discs of *Trichoderma* species were added in the center of the plate after cotton swabbing from the CPG broth of *Ralstonia* spp. For *B. subtilis* and *P. fluorescens*, the line was streaked parallel to the streak of *Ralstonia* spp. in two different CPG agar plates using dual culture technique. After 72 hr of incubation, live microscopic examination on the culture plate was done using a digital microscope (Olympus CX-43, Tokyo, Japan). Images were captured to visualize the interaction of the individual strains of biocontrol agents with *Ralstonia* spp *.*

### Effect of sucrose on the population of biocontrol agents

There is common practice in Nepal of keeping bio-based products in 5–10% (w/v) sucrose solution for 2–4 h before application to the plant. Hence, to evaluate the effect of 5% (w/v) sucrose solution on the cell number (growth) of the biocontrol agents, 1 ml of concentrate containing 1 × 10 ^-9^ cells ml ^-1^ was kept in 5% sucrose solution, made using autoclaved distilled water. Cell count was taken using a Hemocytometer (Reichert, Buffallo, NY, USA) with trypan blue at 1 h, 2 h, 3 h and 24 h to observe the effect on the microbial population.

### Evaluating the effects of biocontrol agents in the field

For the field study, the concentrates containing 10 ^9^ cells/ml of the respective biocontrol agent were used. The densities of the cells were determined using Hemocytometer (Reichert, Buffallo, NY, USA) ^32^. The TSM broth of all six isolated native *Trichoderma* species viz. AA2, AG3, AKD, A5, A9 and A10 were mixed in equal proportion to prepare 1 liter of concentrate containing 10 ^9^ cells/ml. Similarly, two native *P. fluorescens* species viz. PFB and PFS were mixed in an equal proportion to prepare 1 liter of concentrate containing 10 ^9^ cells/ml. Concentrate of native *B. subtilis* viz. BS containing 10 ^9^ cells/ml was used to analyze the effect of *B. subtilis* as a possible biocontrol agent.

Before the application in the tomato plots at Agro Narayani Farm, the prepared concentrates of biocontrol agents were taken to the field and were further diluted at the rate of 2ml/l of tap water containing 5% (w/v) sucrose. After 2 h of incubation in 5% sucrose water, the diluted solutions were applied in the root of tomato plants at the rate of 100 ml per plant. The processes of applications were repeated every 7 days for 8 weeks (total of 8 applications) by preparing fresh dilutions in 5% sucrose solutions 2 h prior to applications. Effects after the 8 weeks of continuous application were measured in the field by identifying the number of plants that underwent recovery after treatment. 6 plots were treated with the biocontrol agent and 2 plots were used as controls. Chemical treatment was done in one plot (positive control plot) using the combination of Agricin (9% Streptomycin Sulphate and 1% Tetracycline Hydrochloride) at the rate of 100 ml of 0.1% (w/v) solution per plant from Agricare Nepal Pvt. Ltd., Nepal and Bavistin (50% carbendazim) at the rate of 0.2% (w/v) solution per plant from Crystal Crop Protection Pvt. Ltd., India. For negative control, no treatment methods were selected in one plot.

The treatment plots were designed such that the effects of the individual biocontrol agent and effects of combination treatment can be studied ( Table 2).

**Table 2.** Design of treatment plot to study the effect of different treatment methods on controlling the bacterial disease. One plot comprised of 100 tomato plants and 8 plots in total were studied; area of 50 m ^2^ per plot.

Plot (100 plants/Plot)	Treatment
**1**	BS ( *Bacillus subtilis*)
**2**	TV ( *Trichoderma* spp. mix)
**3**	G ( *Pseudomonas fluorescens*)
**4**	BS+TV ( *Bacillus subtilis* and *Trichoderma* spp. Mix)
**5**	TV+G ( *Trichoderma* spp. Mix and *Pseudomonas fluorescens*)
**6**	TV+G+BS ( *Bacillus subtilis*, *Trichoderma* spp. Mix and *Pseudomonas fluorescens*)
**7**	No application
**8**	Chemical application (Agricin: 9% Streptomycin Sulphate and 1% Tetracycline Hydrochloride + Bavistin: 50% carbendazim)

### Statistical analysis

Statistical analysis of the data was done using IBM SPSS Statistics (ver 23) and figures and data were made through Microsoft Excel 2007 and Microsoft Word 2007.

## Results

### Weather data

Weather data of Chitwan District for temperature, humidity and rainfall were collected from www.worldweatheronline.com from March 2017 to July 2017 (see Table 3).

**Table 3.** Data on average weather over the study period.

Month	Maximum temperature °C	Minimum temperature °C	Average temperature °C	Rainfall (mm)	Cloud (%)	Humidity (%)
**March**	26	11	21	42.3	16	47
**April**	32	17	27	184	8	43
**May**	32	19	28	319.5	13	54
**Jun**	32	21	28	536	24	71
**July**	30	21	27	791.8	49	84

Source: www.worldweatheronline.com

The bacterial wilt outbreak was reported in the late May, 2017, when the temperature and humidity level increased. Higher temperature and moisture favors the growth of *Ralstonia* spp. ^33^

### Identification of bacterial wilt in the field

From the field examination of the tomato plants, observation revealed that the leaves were flaccid, adventitious roots started to appear on the stem and ooze appeared after dipping the stem in water. Also, field experts from KCC confirmed the presence of bacterial wilt infection, due to their years of experience in plant disease diagnosis.

### Isolation and identification of *Ralstonia* spp.

Infected plant saps from six xylems showed similar bacterial colonies on CPG medium. All the colonies were similar to a virulent type, as the appearance was white or cream-colored, irregularly-round, fluidal, and opaque on CPG medium ^34, 35^. Gram staining and observation using a microscope showed that the bacteria were gram negative, rod-shaped and non-spore forming, which further confirmed that the bacteria was *Ralstonia* spp.

### Evaluating the efficacy of antagonists against *Ralstonia* spp.

Strains tested showed antagonistic effect against *Ralstonia* spp., with inhibition zone radii ranging from 13 to 21.33 mm ( Table 4). *P. fluorescens* PFS isolated from *Parthenium hysterophorus* L. rhizoplane soil was most potent compared to other *P. fluorescens* strains. *Trichoderma virens* ATV and *Trichoderma harzianum* ATH provided by TNAU were the least and most potent species. Among six natively isolated *Trichoderma sp.*, AA2 isolated from *Parthenium hysterophorus* L. rhizoplane soil was most potent and AKD and A9 were least potent. However, the activities of native *Trichoderma* spp. were satisfactory in the term of inhibition zone shown. *Bacillus subtilis* BS isolated from Bhaktapur top soil did not show satisfactory inhibition activity. The complete photographs showing zone of inhibitions can be retrieved from data availability section. 

**Table 4.** Inhibition zone made by the isolates used as biocontrol agents against *Ralstonia* spp. using agar well diffusion technique. All the data are generated using three replications. Values are means (±SE) zone of inhibition (ZOI) in mm against *Ralstonia* spp. (n=3, P <0.05). S30 denotes streptomycin sulphate used at the dilution of 30 mcg. 5 mm diameter of punch hole is included in the data. Code is in reference to Table 1.

Code	ZOI (in mm)
AA2	20.67 ± 0.88
AG3	18.33 ± 0.33
AKD	17.00 ± 0.58
A5	19.67 ± 0.67
A9	17.00 ± 0.58
A10	17.33 ± 0.66
ATH	21.33 ± 0.88
ATV	16.33 ± 0.66
ATA	18.00 ± 0.58
BS	13.00 ± 1.53
PFS	22.33 ± 3.38
PFB	16.33 ± 0.33
PFA	21.00 ± 0.58
S30	37.67 ± 1.33

**Figure 1.**
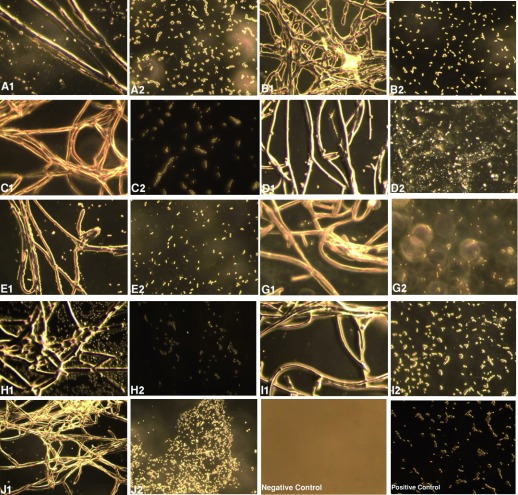
Digital images (400X) of microscopic analysis in dark phase representing the interactions of different *Trichoderma* species against *Ralstonia* spp. A1–J1 shows strains AA2, AG3, AKD, A5, A9, A10, ATA, ATH and ATV, respectively (see Table 1), growing on *Ralstonia* spp., which was cotton swabbed onto CPG agar plates. A2–J2 represents growth of *Ralstonia* spp *.* 4 cm away from the growth of *Trichoderma* spp., viz., A2, AG3, AKD, A5, A9, A10, ATA, ATH and ATV, respectively, on the plate. Negative control (i.e., blank plate without any swabbing) and positive controls (i.e. *Ralstonia* spp. without *Trichoderma* species) are also included.

The effect of biocontrol agents against *Ralstonia* spp. was analyzed at a microscopic level in dark phase using image analyzer (Olympus CX-43, Tokyo, Japan). Figure 1– Figure 3 show the distinct inhibitory effect on the growth and survival of *Ralstonia* spp. caused by different biocontrol agents. From the figures, it can be seen that the most of the pathogenic cells ( *Ralstonia* spp.) were either killed or growth was retarded or limited in or towards the region of growth of antagonists as compared to the region away from the growth of antagonists.

**Figure 2.**
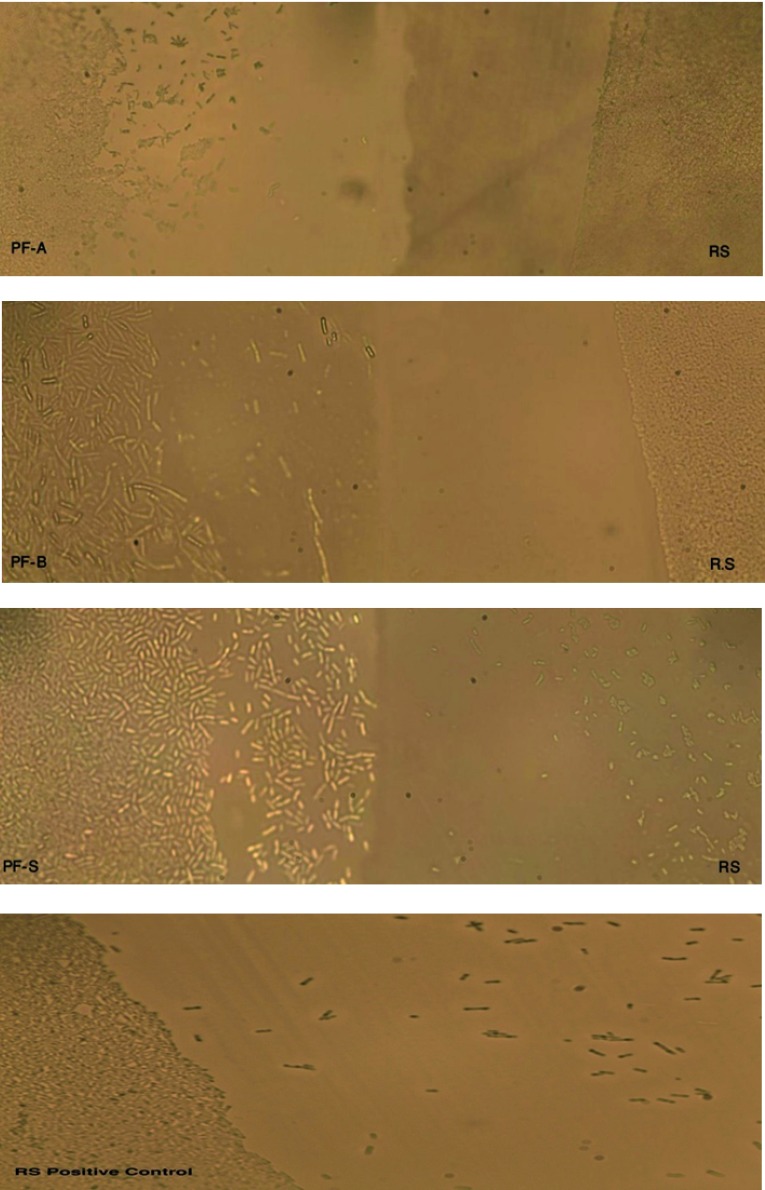
Digital images (400X) of microscopic analysis in bright field representing the interactions of *Pseudomonas fluorescens* species against *Ralstonia* spp. PFA, PFB and PFS represent *P. fluorescens* (PF) species (see Table 1), and RS represents *Ralstonia* spp *.* Both PF and RS were streaked near to each other to see the interaction between the two species.

**Figure 3.**
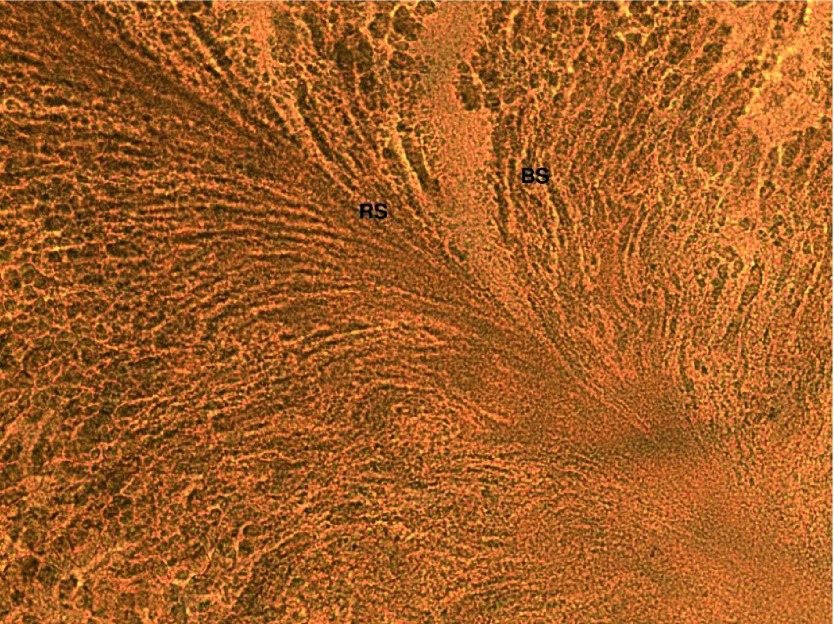
Digital image (400X) of microscopic analysis in bright field representing the interaction of *Bacillus subtilis* against *Ralstonia spp.* BS represents *Bacillus subtilis* species (see Table 1), and RS represents *Ralstonia* spp *.* Both BS and RS were streaked near to each other to see the interaction between two species. BS has completely overgrown the RS streak on the CPG agar plate, which suggests that there has been an interaction between RS and BS and BS is dominant over RS.

The digital images from Figure 1 reveal that the population of *Ralstonia* spp. is significantly less and most of the cells are dead in the region of growth of samples treated with *Trichoderma* spp., compared to the region 4 cm far from the growth of *Trichoderma* spp as the *Ralstonia* is a rod-shaped bacteria but near the growth region of *Trichoderma*, most of the bacterial cells clearly seems to be round and distorted in shape with much lower population density than the region far away This indicates the fact that the *Ralstonia* cells that got inoculated in the plate during the cotton swab were unable to multiply and grow in the zone where *Trichoderma* was growing.

The images from Figure 2 reveal that PF tended to grow on the side of RS, whereas RS tended to restrict the growth towards the PF species. RS positive control (without PF streak nearby) tended to spread, which confirms the spreading pattern of RS. The microscopic study was to verify a fact that the incompatible species does not grow towards each other and the growth of dominant microbes always surpasses the growth of other recessive microbes. The same phenomenon was observed in the microscopic analysis as *Ralstonia* spp. formed a clear boundary or showed restricted spreading in the region of the streak as compared to the Positive control but the streak of *Pseudomonas fluorescence* was easily growing towards the *Ralstonia spp*. 

### Effect of sucrose on the population of biocontrol agents

**Figure 4.**
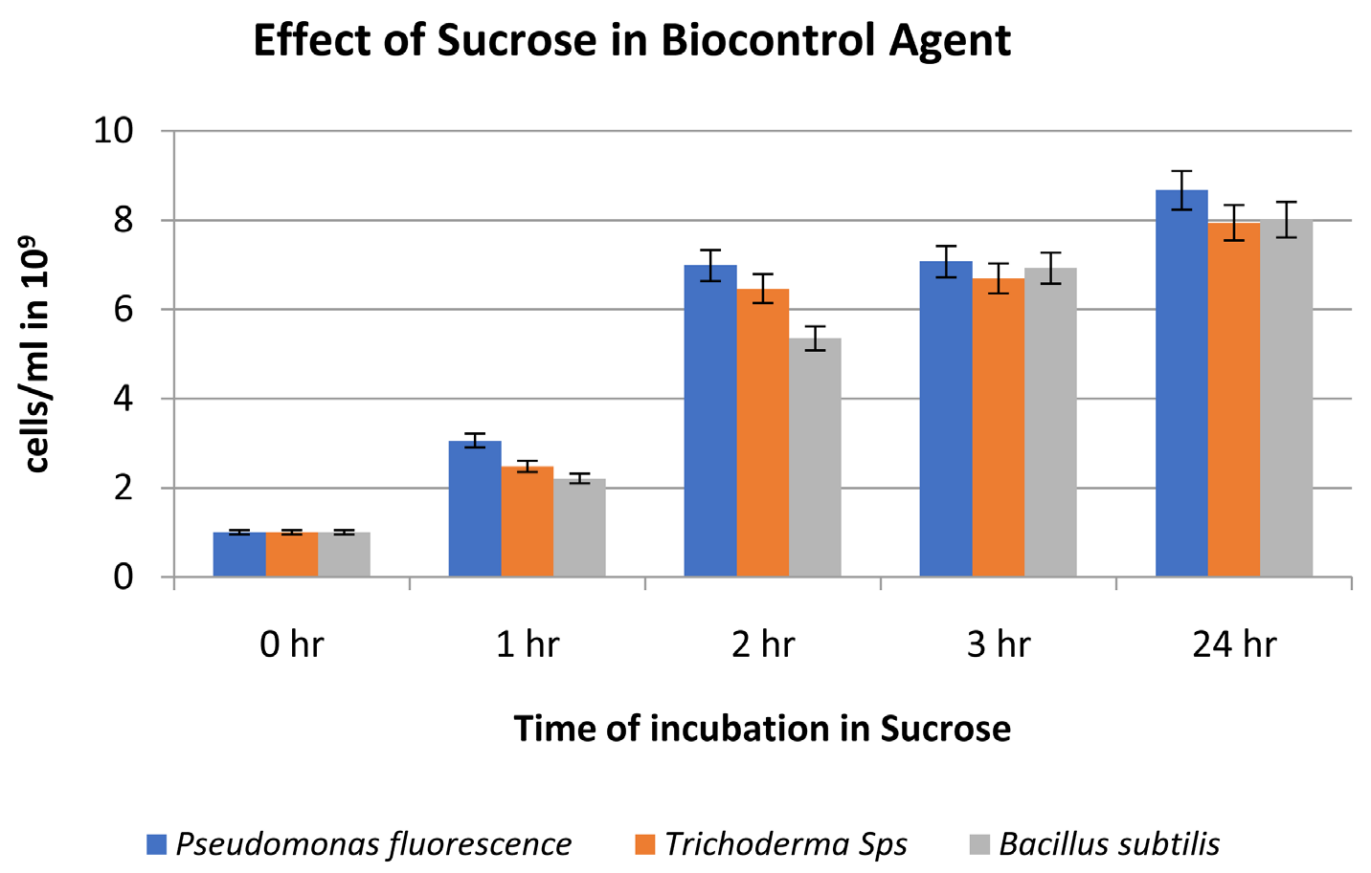
Effect of sucrose on the growth of *Pseudomonas fluorescens*, *Trichoderma* spp. and *Bacillus subtilis*. An increase in a number of cells of biocontrol agents was seen over time when these agents were kept in 5% (w/v) sucrose solution (n=3). The error bar represents the 5% error in independently performed experiments. The complete data are available in data availability section of this manuscript.

The effect of sucrose (5% w/v) in the concentrate mixture of individual biocontrol agents was analyzed ( Figure 4), which showed that there was a profound increase in the number of cells of biocontrol agents after 2h of incubation compared with the initial population. The cell count between 2h and 3 h of incubation was not significant as compared with 2 h and 24 h of incubation. Thus, 2 h of incubation in sucrose solution can be considered as optimal time, as lengthier time can result in the growth of contaminant in the solution whilst using tap water in the field.

### Evaluating the effects of biocontrol agents in the field

Before applications, 2ml of respective biocontrol agents of (10 ^9^ cells/ml) were incubated in 5% (w/v) of sucrose solution and incubated for 2 hr. The prepared dilution was thus applied at the rate of 100 ml per plant in the root region every week. 8 applications were done over 8 weeks. 8 plots (100 plants/plot) were selected, out of which one plot was used as positive control/chemical treatment plot (Agricin+Bavistin) and one plot as negative control/zero treatment plot (no treatment given).

**Figure 5.**
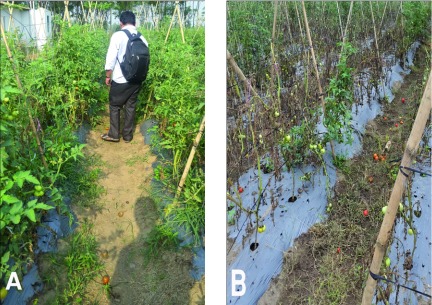
Differences in the field between treated and untreated plots. Field images clearly reveal that the treatment with biocontrol agents has helped to eliminate the bacterial wilt disease in the field after 8 weeks of application. ( **A**) shows the growth and vigor of plants treated with biocontrol agents; ( **B**) shows growth and severity of infection occurred in the untreated plot.

The results are displayed in Table 5 and show that the application inhibited the bacterial wilt infection ( *Ralstonia* spp.) in tomato plants by and the highest rate of plant recovered was 97% from treatment using antagonists, which was comparable with the plant recovery of 94% using the chemical treatment (Agricin and Bavistin). Only 37% of plants were recovered in the plot where no treatment methods were applied. Field Images ( Figure 5) show a clear visualization of growth of plants and severity of infection in treated and untreated plot. The complete photographs of the field trial can be retrieved from the data availability section of this manuscript.

**Table 5.** Effect of applying biocontrol agents to tomato plants infected with bacterial wilt. There was a significant recovery of plants using a mixture of *Trichoderma* species and *Pseudomonas fluorescens*, and the recovery rate was higher than that of chemical treatment. *Bacillus subtilis* did not show significant recovery rate.

S.N.	Treatment plot (100 plants per plot)	No. of plants recovered	No. of plants infected	% recovery
1	BS	84	16	84
2	TV	92	8	92
3	G	96	4	96
4	BS+TV	95	5	95
5	TV+G	97	3	97
6	TV+G+BS	97	3	97
7	Positive control (chemical treatment)	94	6	94
8	Negative Control (w/o treatment)	37	63	37

## Discussion

This research covers the results of the effectiveness of native biocontrol agents in both laboratory and field settings. This research also provides the application strategies of biocontrol agents at the field level. Also, from literature reviews ^10– 17^, it has been shown that these biocontrol agents can be used to control various other bacterial and fungal diseases, such as Fusarium wilt beside bacterial wilt. Hence, application of these biocontrol agents can also help to prevent other diseases in various crops.

The present results, using microscopy, showed that different species of *Trichoderma* and *Pseudomonas fluorescens* clearly hinders the growth of *Ralstonia* spp., which causes bacterial wilt in tomato plants. *Bacillus subtilis* did not show a significant hindrance. *Trichoderma* spp. secret different compounds against bacteria and also produce various secondary metabolites that promote plant growth and yield ^36– 38^. *P. fluorescens* produces various compounds that suppress the growth of *Ralstonia* spp. and also induces systemic resistance in the plant ^39– 41^. *B. subtilis* is well known to induce systemic resistance in plants by secreting various kinds of lipopeptides and secondary metabolites, and this agent also improves plant growth ^42– 44^. Analysis of data from research, field trials and scientific journal reviews suggests that the application of *B. subtilis* may not immediately show results, but a continuous application of this strain in the agricultural field will slowly induce resistance of plants against pathogenic diseases ^42^. Thus, evidence from scientific research show that *Trichoderma* spp. and *P. fluorescens* are effective biocontrol agents against bacterial wilt in compared to *Bacillus subtilis*. 

Pre-application of biocontrol agents can successfully prevent the disease attack ^45^, induce systemic disease resistance in plants and increase the yield from secondary metabolites secreted by the beneficial bacteria in the biocontrol agent. Thus, we recommend that farmers should continuously apply biocontrol agents in their field so that systemic resistance can get induced in plants and also the plants get protected from invasion of pathogens. Although the cost of production get increased but the farmers can sell their products by considering them as IPM (Integrated Pest Management) product or as an organic product, giving better monetary value for the farmers and health benefits for consumers.

From the results, 2 h incubation of the biocontrol agent in 5% (w/v) sucrose solution was judged as suitable practice being carried out in Nepal for application of biocontrol agents due to two facts. First, it was seen that number of cells of biocontrol agents were increased during the incubation period as observed in Figure 4. Second, the water farmers use for drip irrigation generally is unsterilized and comes from an underground source, which may promote the growth of contaminants in sucrose solution if kept for longer periods.

*Trichoderma* spp. and *Pseudomonas fluorescens* provide better results in controlling bacterial wilt in tomato. *Bacillus subtilis* did not perform well in the immediate control of disease. Data from Table 5 reveals that biocontrol agents can be used as the sole method to control bacterial wilt, and the use of chemical methods can be avoided in the field. Also, combination therapy using both *Trichoderma* spp. and *P. fluorescens* seems to be more effective than treatment using each individual biocontrol agent. A 97% control rate was achieved using combination treatment in the field.

For decades, microbiologists have identified pathogen through the use of phenotypic methods ^46^. We authors agree with the fact that 16S rRNA sequencing should be done to identify to the species level for all the microbes used in this manuscript but due to unavailability of the sequencing service in low-income country like Nepal and several unclear quarantine policies in Nepal, researchers in Nepal can only identify to the genus level of microbes by phenotypic methods. If the species has recently diverged then 16S rRNA sequencing alone will not be adequate to assign a species rather it needs additional analytical procedures like sequencing protein coding genes or the intergenic spacer region of the ribosomal gene complex ^46^.

## Conclusions

In the present study, *Trichoderma* spp. and *P. fluorescens* seem to be the best biocontrol agents in controlling bacterial wilt induced by *Ralstonia* spp. The zone of inhibition shown by the various antagonists reveals that native isolates were successful in inhibiting the growth of *Ralstonia* spp *.* The digital microscopy also supports the antagonistic effects of the native isolates.

Also during field application, mixing with 5% (w/v) of sucrose solution and keeping it for 2 h seems to be an effective strategy in better management of bacterial wilt. The application strategies of biocontrol agents with the rate of 100 ml per plant per week successfully recovered the plants from the attack of the pathogen. However, the application rate and amount of biocontrol agents can be varied according to disease severity. Also, the application of the multiple numbers of biocontrol agents can be performed to achieve better results. Hence, native isolates of *Trichoderma spp.* and *Pseudomonas fluorescens* can be used as biocontrol agents to control the bacterial wilt and combined application of these beneficial microbes as bioantagonist can give better results in controlling bacterial wilt infection by *Ralstonia* spp. Results shown by *Bacillus subtilis* were not significant but the scientific researches shows that it can induce systemic resistance in plant with time

## Data availability

The data referenced by this article are under copyright with the following copyright statement: Copyright: © 2018 Yendyo S et al.

Data associated with the article are available under the terms of the Creative Commons Zero "No rights reserved" data waiver (CC0 1.0 Public domain dedication). 

OSF: **Raw values of zone of inhibition by antagonist against *Ralstonia* spp.** ZOI is shown in mm and the data were used for statistical analysis. http://doi.org/10.17605/OSF.IO/9TQCE ^47^

OSF: **Raw values of the 5% sucrose treatment.** The average of these values in cells/ml was taken to create the data in the manuscript. http://doi.org/10.17605/OSF.IO/Q8FVU ^48^

Figshare: **Images of the plates showing the zone of inhibition by different antagonists against *Ralstonia* spp.** Clear zone of inhibition obtained by agar well diffusion technique indicates the bioefficacy of the selected bioantagonists against Ralstonia spp. https://doi.org/10.6084/m9.figshare.5562058.v3 ^49^

Figshare: **Raw digital images (400X) of microscopic analysis representing the interactions of different antagonist against** *Ralstonia* spp. Collection of raw images obtained from digital microscopy in both dark field and bright field microscopy at 400 X zoom. https://doi.org/10.6084/m9.figshare.5561968.v2 ^50^

Figshare: **Field Images of plot design showing pictures of before the treatment and effects after the treatment.** There is significant decrease in the occurrence of disease for the treated plots whereas the bacterial wilt has severely affected in the untreated plot. https://doi.org/10.6084/m9.figshare.5562373.v2 ^51^

Data are available under the terms of the Creative Commons Attribution 4.0 license (CC-BY 4.0).
