# Supplementary material for: Evaluation of Trichoderma spp., Pseudomonas fluorescens and Bacillus subtilis for biological control of Ralstonia wilt of tomato
Source: F1000Res. 2018 Mar 22;6:2028. Originally published 2017 Nov 20. [Version 3] doi: 10.12688/f1000research.12448.3 (PMC5854981; doi:10.12688/f1000research.12448.3)
Supplement: Peer review history

# Peer review: Referee response for version 2

**DOI:** 10.5256/f1000research.15184.r31044

**Published:** 2018-03-14

**Authors:** Teresa A. Coutinho

**Affiliations:** 1 Department of Biochemistry, Genetics and Microbiology, Centre for Microbial Ecology and Genomics, Forestry and Agricultural Biotechnology Institute, University of Pretoria, Pretoria, South Africa

"All the colonies were similar to avirulent type, as the appearance was white or cream-colored, irregularly-round, fluidal, and opaque on CPG medium" - if they were avirulent i.e. incapable of causing a disease, then how did bacterial wilt occur  in the field? 

 I suggest you exclude the last paragraph of the discussion concerning access or rather lack of access to a sequencing platform. To overcome this obstacle, researchers in low-income or developing countries should collaborate with those in the developed world (my opinion).

I have read this submission. I believe that I have an appropriate level of expertise to confirm that it is of an acceptable scientific standard.

# Response

**Published:** 2018-03-14

**Authors:** Binayak Raj Pandey

**Affiliations:** Agricare Nepal Pvt. Ltd., Nepal

Dear Prof. Dr. Teresa A. Coutinho,

 We are very honored that our manuscript was evaluated by you who have published the first report of bacterial wilt caused by *Ralstonia solanacearum* on eucalypts in South Africa and who know the pathogen in detail. Professor, we are really honored !!

 You have provided us the insight that how can a research manuscript in a particular topic be polished to a level of scientific readability and acceptance. Your comments in the first version were extremely helpful and i will keep those suggestions in the heart so that i can perform a better scientific research. 

 Regarding, ' **avirulent strain**', it is a typo and it was meant to be " **a virulent**" and hope it will be corrected, thank you for pointing out :-). 'Virulent' and 'Non-Virulent' strains will be more suitable terms to distinguish between them (I think).

 I would like to keep the point concerning access or rather a lack of access to a sequencing platform in low-income country like Nepal as this is a fact and somebody may site it too. We are in an era where Human DNA is being sequenced but in countries like Nepal even the simple microbial sequencing service is not available not even with government and universities. I am not criticizing but this is the situation which we have to accept and act on it for improvements. This is a major reason why researchers have to go to foreign countries to get trained and hands-on-experience for such. Most of the institutions and universities in Nepal are ready to conduct a collaborative research with researchers in developed countries but i think Nepal is not able to seek the attention of researchers around the globe despite being rich in biodiversity. These kinds of initial research may attract researchers to Nepal (I hope so). I would like to share an experience with an Indian Prof. Dr. S. Nakeeran from Tamil Nadu Agricultural University, he quoted that **soil of Nepal is so rich in microbial load that it needs more dilution in order to isolate the desired species**.

 It was a challenge for me to publish an acceptable manuscript with limited technologies available in Nepal. Thanks to F1000Research for eliminating the Article Processing Charges for this manuscript, without which we could not have published in this platform. 

 Thank you again Professor and F1000Research team for your ideas and support.

 With Regards,

 Binayak Raj Pandey

# Peer review: Referee response for version 1

**DOI:** 10.5256/f1000research.13479.r28139

**Published:** 2018-02-09

**Authors:** Dhurva P. Gauchan

**Affiliations:** 1 Department of Biotechnology, School of Science, Kathmandu University, Kathmandu, Nepal

This manuscript contributes data to the biological control of Bacterial wilt of tomato using microbial consortium which can be turned into commercial product in Nepal. The work is well planned, the methods seem adequate, the results are in a non-negligible amount and discussed on the basis of a convenient literature set. But, the English used in the paper needs to be corrected. If author/s are not expert, they can take help from English expert to rewrite the Sentences. I believe that the manuscript is worthy of being indexed and that it could be accepted after a minor revision, for which I give now some comments, with the aim of helping the authors in obtaining a definitely acceptable version. 

**Title:**

 Change the title

 Evaluation of *Trichoderma spp*. and *Pseudomonas fluorescence* for biological control of Bacterial wilt of tomato. 

 As Bacillus results are not satisfactory the author supposed to concentrate on only one bacterial and fungal species. 

**Abstract:**

 Background: *Ralstonia solanacearum* is the major bacterial disease in tomato, which invades the roots of diverse plant hosts and colonizes xylem vessels causing wilt, especially in tropical, subtropical and warm-temperate regions. 

 Rewrite the sentence: *Ralstonia solanacearum* is the major bacterial disease ( *Ralstonia solanacearum* is the not the disease, it is causal organism) 

 Results: Incorporate the findings of *Bacillus subtilis* (Remove if findings are not up to the mark)

 Conclusion: Rewrite the conclusion as it gives confusing information.  

**Introduction:**

 This study focuses on evaluating the efficacy of different native isolates of *Trichoderma* species, *B. subtilis* and *P. fluorescence* against bacterial wilt disease caused by the pathogen *R. solanacearum* in the tomato plant. The study hypothesizes that the native isolates of *Trichoderma* spp., *B. subtilis* and *P. fluorescence* can be used as bioantagonistic agents to control bacterial wilt ( *R. solanacearum*) of tomato. This study tries to reflect the bioantagonistic effects by the microscopic examinations, agar well diffusion technique and by applying the concentrate of biocontrol agents in tomato plots, to calculate their efficiency by comparing them with chemical methods of treatment. 

 Rewrite and give stress to firm objectives, sentences are poorly written need more accurate writing.  

**Methods:**

 Write the Latitude: 27.582016 and Longitude: 84.272259 in scientific manner. Trial design is not described (Field Experimentation: Experimental Design). Weather data is not needed. Table: 1 Write full scientific name of plants with authority. Follow strictly ICBN system wherever plant name is used.  

 Briefly, the stems of the infected plant were washed three times with autoclaved distilled water and then blot dried.

 Omit Briefly 

 Table: 1 Scientific names of source are not uniform. Write complete scientific name of plants with authority. What is the scientific reason to collect samples all from tropical region except Bacillus which is collected from mid hill regions of Nepal. Justify.   

 In this section: Effect of sucrose on the population of biocontrol agents: quote supportive references for this methods. 

 Choose appropriate statistical tools to evaluate your generated data. Used statistical tools are insufficient.

**Results:**

 Only 4 months weather data is not justifiable until it is compared for two consecutive years. Better skip this data which does not show relevance with your work.  

*Bacillus subtilis* BS isolated from Bhaktapur top soil did not show satisfactory inhibition activity. When does Bacillus give not satisfactory results then better omit Bacillus content from the paper.

 Table 4. Inhibition zone made by the isolates used as biocontrol agents against *Ralstonia solana* *cearum *using agar well diffusion technique.

It is very nice if some supportive pictures are added in this section.

**Discussion:**

 The present results, using microscopy, showed that different species of *Trichoderma* , *Pseudomonas fluorescence* and *Bacillus su btilis* clearly hinders the growth of *Ralstonia solanacearum* , which causes bacterial wilt in tomato plants.

 AND 

 Results: *Bacillus subtilis* BS isolated from Bhaktapur top soil did not show satisfactory inhibition activity.

 Both statements are contradictory. Justify

 Make a common consensus and write obtained results clearly.

I have read this submission. I believe that I have an appropriate level of expertise to confirm that it is of an acceptable scientific standard.

# Response

**Published:** 2018-02-17

**Authors:** Binayak Raj Pandey

**Affiliations:** Agricare Nepal Pvt. Ltd., Nepal

Dear Dr. Dhurba Gauchan Sir,

 The comments were extremely helpful to realize few shortcomings of the manuscript. We have briefly detailed and corrected few abnormalities in Abstract, Introduction, Methods, Results and Conclusion sections of the manuscript. Your suggestions has definitely taken the manuscript to next level.

 Bacillus subtilis did not show satisfactory results and we have added few points to make it clear that the results were not satisfactory. We have added the clarification of why the Bacillus subtilis was taken from Bhaktapur. The other research articles and our observations (not mentioned in this research) suggest that Bacillus subtilis can induce systemic resistance in a plant with long-term use. Also, the scientific names of the plants were added with authority in version 2.

 From next research onwards we will try to incorporate the ideas and suggestions from this manuscript and make also record the weather data for two consecutive years. It's a challenge to work with Biocontrol agents in Nepal as very few genetic tools are available with no option for sequencing and we have to depend on the 3rd world for such services which possess the risk of native potent strains being claimed by others. Hope this lack will end soon in Nepal. 

 Only few scientist are working in the field of Biocontrol agents and organic farming in Nepal and we are honored that you who fall among those scientist have reviewed our article. 

 Regards,

 Binayak Raj Pandey

# Peer review: Referee response for version 1

**DOI:** 10.5256/f1000research.13479.r30061

**Published:** 2018-02-05

**Authors:** Teresa A. Coutinho

**Affiliations:** 1 Department of Biochemistry, Genetics and Microbiology, Centre for Microbial Ecology and Genomics, Forestry and Agricultural Biotechnology Institute, University of Pretoria, Pretoria, South Africa

The manuscript, particularly the introduction and discussion is poorly written. Each paragraph is an entity on its own and should contain one topic, with a introductory, body and concluding sentence. Many of the paragraphs contain too many topics.

 Please correct the spelling of Pseudomonas fluorescens throughout the manuscript.

 In the abstract the authors mention that R. solanacearum produces several toxins – toxins are not the major virulence factor in this pathogen – why this sentence was included in the abstract and no where else in the manuscript, is difficult to undertand.

 The use phenotypic methods to identify bacteria is no longer sufficient. None of the biocontrol agents or even the causal agent of the wilt symptoms observed where appropriately identified in this study. Based on the latest publications - two Ralstonia species can cause bacterial wilt - R. solanacearum and R. pseudosolanacearum. So which one were they working with? How where the references strains of the biocontrol agents used? Not clear - again their identity needs to be verified by sequencing at the very least (of 16S rRNA).  

 It is unclear how the inoculum concentrations of the bacterial control agents were determined.

 In the experimental plot the authors mention that bacterial wilt was present the previous year. Although I would agree that the pathogen would be present the following year, how did they insure that all areas of the experimental plots were contaminated with the pathogen? Was there 100% loss the previous year?

 The use of sucrose in the preparation of the biocontrol agents is concerning. Although the authors noted that it is a common practice in Nepal, the reasons why they would do this need to be provided.

 The Figures 1-3 are not very useful. I am not exactly sure what the authors are trying to show. Also, in Figure 1 how do your know the bacterial cells are dead?

 There are no error bars on Figure 4.

 In Table 4 what do the authors mean by variance.

 The discussion and conclusions should be reworked into a comprehensive section. Currently the conclusions are longer than the discussion which is not correct.

I have read this submission. I believe that I have an appropriate level of expertise to state that I do not consider it to be of an acceptable scientific standard, for reasons outlined above.

# Response

**Published:** 2018-02-07

**Authors:** Binayak Raj Pandey

**Affiliations:** Agricare Nepal Pvt. Ltd., Nepal

Dear Dr. Teresa A. Coutinho,

Thank you for your creative comments. Your comments have helped us to add few points to make the manuscript more clear. I have tried to address most of the issues in version 2 of the manuscript.

1. Regarding the issue- **The manuscript, particularly the introduction and discussion is poorly written. Each paragraph is an entity on its own and should contain one topic, with an introductory, body and concluding the sentence. Many of the paragraphs contain too many topics.**

-The introduction and discussion section has been re-written. English language has already been reviewed and corrected by native speakers.

2. Regarding the issue- **Please correct the spelling of Pseudomonas fluorescens throughout the manuscript.**

-The spelling of Pseudomonas fluorescens throughout the manuscript has been corrected. We found some authors using previous spelling as cited in Reference 11 of the manuscript.

3. Regarding the issue- **In the abstract, the authors mention that R. solanacearum produces several toxins – toxins are not the major virulence factor in this pathogen – why this sentence was included in the abstract and no where else in the manuscript, is difficult to understand**

-In the abstract, the statement that R. solanacearum produces several toxins has been replaced by virulence factors. Thanks for the indication.

4. Regarding the issue- **The use phenotypic methods to identify bacteria is no longer sufficient. None of the biocontrol agents or even the causal agent of the wilt symptoms observed were appropriately identified in this study. Based on the latest publications - two Ralstonia species can cause bacterial wilt - R. solanacearum and R. pseudosolanacearum. So which one were they working with? How where the references strains of the biocontrol agents used? Not clear - again their identity needs to be verified by sequencing at the very least (of 16S rRNA). ** 

-R. solanacearum has been replaced by Ralstonia spp. to address the fact that the bacterial wilt was present but which species of Ralstonia has caused it needs genetic services to identify. I agree that 16S rRNA is a recently evolved tool and Microbiologist are using Phenotypic methods to identify the microbes from decades. Microbiological tool is still a valid method to identify the microbes to genus level and is still being used as a tool in hospitals, pathology labs and research centers to do so where human life is involved and 16S rRNA is used to identify to the species level. Nepal being a low-income country don't have any sequencing service in any of government or private organization till the date. Hence, the issue with species-level identification has been added in the "Discussion" section of the manuscript and throughout the manuscript, we have mentioned only the genus level of the microbes except Pseudomonas fluorescens as they both can be distinguished easily by morphological and microscopic examination and fluorescence that they emit when subjected to UV light. As far as Bacillus subtilis is concerned I have mentioned the GeneBank Accession Number for it where I am the first author.   

-The wilt symptoms are clearly mentioned and cited properly in the **"**Isolation and identification of Ralstonia **" **subheading of "Methods section" in the manuscript.

- I have now clearly mentioned that How where the references strains of the biocontrol agents used? in "Isolation of antagonists" sub-heading of "Methods" section.

5. Regarding the issue- **It is unclear how the inoculum concentrations of the bacterial control agents were determined.**

-It has clearly been mentioned in "Evaluating the effects of biocontrol agents in the field" subheading in "Method Section" that a Hemocytometer was used. I don't think we need to mention the protocol of using Hemocytometer to count the cells as it is a very simple procedure adopted even in the diploma curriculums and re-writing such simple protocols will make the manuscript a mesh and will make the readers diverted from the main contents in the manuscript.     

6. Regarding the issue- **In the experimental plot the authors mention that bacterial wilt was present the previous year. Although I would agree that the pathogen would be present the following year, how did they insure that all areas of the experimental plots were contaminated with the pathogen? Was there 100% loss the previous year?**

-We did not mention the word "previous year" in any section of the manuscript rather we mentioned "Previous Harvest". In Nepal Harvest can be done 2 times in a year due to pleasant climatic conditions. I have addressed the issue by mentioning that the last harvest was done 3 month before the test started and there was heavy loss in the crop due to wilt infection. We have seen the case that even after 2 years there has been re-occurrence of the wilt in the field, its just 3 months, I think we can buy the fact that the pathogens were present in the field also we tested the soil for presence of pathogen and i have added the same in the manuscript. No, the loss was not 100 % as it has clearly been indicated in Table 5 that with the Negative control (w/o any treatment) there was 63 % loss in the plot. I agree that we should have injected bacterial wilt in the field but it went against our ethics to deliberately destroy the crop of the poor farmer. Hence unlike any other research paper where only field data is taken into account to evaluate the efficiency by injecting the pathogen into the field we thought to evaluate the efficacy in the lab and then comparing the validity of the data from the field. We in the research paper have clearly indicated that the evaluation in the field was done in a natural environment giving all the necessary fertilizers as needed without adultering the pathogen population in the soil where the previous infection occurred. The distance between the treated and untreated plots were merely 0.5 meter in length hence it seems impossible that the pathogens cannot effect the nearby plot. If you seen the pictures from data availability section of the manuscript then you can clearly note that the plot where Bacillus subtilis was given as treatment method was effected by the wilt (16 % infection) whereas the infection was 8 % in the plot that was merely 0.5 m from the BS treatment plot. The Negative Control plots (w/o Any Treatment) was also merely 0.5 m from all chemically treated plot but the infection rate was 63 % for Negative Control Plots and 6 % for chemically treated plot. Is that even possible if the pathogen was not present in the soil ?    

7. Regarding the issue- **The use of sucrose in the preparation of the biocontrol agents is concerning. Although the authors noted that it is a common practice in Nepal, the reasons why they would do this need to be provided.**

-We have now clearly mentioned and restated in a single paragraph in "Discussion" Section. We just wanted to share our experience that if followed for commercial formulations of Biocontrol Agents gives a better result than just directly spreading it in the field. The other farmers & scientists in another country can adopt the same practice and share their outputs of the trials. The practice that has been mentioned in the paper merely concern the output that paper is trying to give.

8. Regarding the issue- **The Figures 1-3 are not very useful. I am not exactly sure what the authors are trying to show. Also, in Figure 1 how do your know the bacterial cells are dead?**

- We have now clarified in detail in "Result" section that how the bacterial cells were assumed to be dead and what we are trying to show.

9. Regarding the issue- **There are no error bars in Figure 4.**

-We have changed the style of the graph and also added the error bars. The detail data can be found in the "Data Availability" Section.

10. Regarding the issue- **In Table 4 what do the authors mean by variance.**

- We have removed the variance column, we thought that the variance data could be useful to some statisticians. The detail data can be found in the "Data Availability" Section.

11. Regarding the issue- **The discussion and conclusions should be reworked into a comprehensive section. Currently, the conclusions are longer than the discussion which is not correct**.

-We have reworked both the conclusion and discussion section and made discussion longer than the conclusion.

There are a lot of manuscripts being published on microbes without 16S rRNA sequencing results and we should consider the fact that in some cases 16S rRNA sequencing alone is not sufficient to identify the species. We will try to identify the species in future manuscripts and if the species are novel then we will be happy to publish the results of that too.

Hope I have addressed the major issues in this manuscripts and I assure you that the research from the beginning has been extensively monitored by the professionals from USAID and the manuscript has been reviewed by agricultural scientists from Nepal and USA as mentioned in the manuscript itself at the beginning. I think you can easily understand that it's a challenging work when the biocontrol agents come and the interactions are complex with the pathogens as you have already worked with Ralstonia and we have also cited your work in Reference 25 of our manuscript. Hence this manuscript is just a preliminary work to give light on how the interaction can be and such research in Nepal can promote the use of Biocontrol Agents in Nepal and we are continuously thriving to establish the use of these beneficial microbes by working with USAID, Winrock International and FAO in Nepal. The results that we are observing from the fields are astonishing and hope we will soon publish more articles on biocontrol agents where genetic works are done. 

With Regards,

Binayak Raj Pandey
